# Supplementary material for: Comparative genomics provides new insights into the diversity, physiology, and sexuality of the only industrially exploited tremellomycete: Phaffia rhodozyma
Source: BMC Genomics. 2016 Nov 9;17:901. doi: 10.1186/s12864-016-3244-7 (PMC5103461; doi:10.1186/s12864-016-3244-7)
Supplement: Additional file 6: — List of orphan genes with links to PFAM (related to Additional file 1: Table S1). (ZIP 1428 kb) [file 12864_2016_3244_MOESM6_ESM.zip › BLAST_HTML_FTR/G00132_P.html]

BLAST Search Results


```
BLASTP 2.2.27+


Reference:
Stephen F. Altschul, Thomas L. Madden, Alejandro A. Schäffer,
Jinghui Zhang, Zheng Zhang, Webb Miller, and David J. Lipman (1997),
"Gapped BLAST and PSI-BLAST: a new generation of protein database
search programs", Nucleic Acids Res. 25:3389-3402.


Reference for
composition-based statistics:
Alejandro A. Schäffer, L. Aravind, Thomas L. Madden, Sergei
Shavirin, John L. Spouge, Yuri I. Wolf, Eugene V. Koonin, and
Stephen F. Altschul (2001), "Improving the accuracy of PSI-BLAST
protein database searches with composition-based statistics and
other refinements", Nucleic Acids Res. 29:2994-3005.


Database: nr
           71,551,133 sequences; 26,053,659,533 total letters


Query= G00132_P

Length=1036
                                                                      Score     E
Sequences producing significant alignments:                          (Bits)  Value

emb|CED83977.1|  hypothetical protein [Xanthophyllomyces dendrorh...  1004    0.0  
emb|CED83978.1|  hypothetical protein [Xanthophyllomyces dendrorh...   614    0.0  
gb|KIR41283.1|  hypothetical protein I313_02403 [Cryptococcus gat...  44.3    0.71 
gb|KIR26362.1|  hypothetical protein I309_04696 [Cryptococcus gat...  44.3    0.72 
gb|KIR69972.1|  hypothetical protein I310_06292 [Cryptococcus gat...  44.3    0.75 
gb|KIR34330.1|  hypothetical protein I352_03574 [Cryptococcus gat...  44.3    0.76 


 >emb|CED83977.1| hypothetical protein [Xanthophyllomyces dendrorhous]
Length=504

 Score = 1004 bits (2597),  Expect = 0.0, Method: Compositional matrix adjust.
 Identities = 503/504 (99%), Positives = 503/504 (99%), Gaps = 0/504 (0%)

Query  532   MSSDRLMSRSFWIFYLGLTIELHETLTSCFWNGMEDLVRTPPFVRLNHLIHRESSAEHAD  591
             MSSDRLMSRSFWIFYLGLTIELHETLTSCFWNGMEDL RTPPFVRLNHLIHRESSAEHAD
Sbjct  1     MSSDRLMSRSFWIFYLGLTIELHETLTSCFWNGMEDLARTPPFVRLNHLIHRESSAEHAD  60

Query  592   GIENMLGALGELGAGLMSLIDSILKIIIHGLSPSSRSQSPSFTSGRSKIDHLVETKTTNS  651
             GIENMLGALGELGAGLMSLIDSILKIIIHGLSPSSRSQSPSFTSGRSKIDHLVETKTTNS
Sbjct  61    GIENMLGALGELGAGLMSLIDSILKIIIHGLSPSSRSQSPSFTSGRSKIDHLVETKTTNS  120

Query  652   AIIRATQNAISTFVRAELFSDLLSTNREDVGLIDSEAERLKSGLVFSKATSLCVLNQLTS  711
             AIIRATQNAISTFVRAELFSDLLSTNREDVGLIDSEAERLKSGLVFSKATSLCVLNQLTS
Sbjct  121   AIIRATQNAISTFVRAELFSDLLSTNREDVGLIDSEAERLKSGLVFSKATSLCVLNQLTS  180

Query  712   LSDRREDGNEIRHEGEEERIGLVLRTIIDYALGAGAEKAREIVGTIIGRLIPDPPFSSLS  771
             LSDRREDGNEIRHEGEEERIGLVLRTIIDYALGAGAEKAREIVGTIIGRLIPDPPFSSLS
Sbjct  181   LSDRREDGNEIRHEGEEERIGLVLRTIIDYALGAGAEKAREIVGTIIGRLIPDPPFSSLS  240

Query  772   STVSTSNSSSIGWGAWDRILYDNSIHMSFLILILTSTSPSIQPFFNKIRLFPSLSTKSFD  831
             STVSTSNSSSIGWGAWDRILYDNSIHMSFLILILTSTSPSIQPFFNKIRLFPSLSTKSFD
Sbjct  241   STVSTSNSSSIGWGAWDRILYDNSIHMSFLILILTSTSPSIQPFFNKIRLFPSLSTKSFD  300

Query  832   LSSPSLSPSVLQTNDDDVGVELVGKASSTSILEWMFASPTSSLDTAIGSISQTTSIITSN  891
             LSSPSLSPSVLQTNDDDVGVELVGKASSTSILEWMFASPTSSLDTAIGSISQTTSIITSN
Sbjct  301   LSSPSLSPSVLQTNDDDVGVELVGKASSTSILEWMFASPTSSLDTAIGSISQTTSIITSN  360

Query  892   PLDGPISSIVPLAPTMTTTSTMTATAVPTPVGTMAAATVGGMLSVPIQRTSKTYRQNEFR  951
             PLDGPISSIVPLAPTMTTTSTMTATAVPTPVGTMAAATVGGMLSVPIQRTSKTYRQNEFR
Sbjct  361   PLDGPISSIVPLAPTMTTTSTMTATAVPTPVGTMAAATVGGMLSVPIQRTSKTYRQNEFR  420

Query  952   SSRSSTLTLPPGAPLTVSPDPSGTVLGGSIGPGGGLGVGMIGSGGVVQKVNASRAPSKHV  1011
             SSRSSTLTLPPGAPLTVSPDPSGTVLGGSIGPGGGLGVGMIGSGGVVQKVNASRAPSKHV
Sbjct  421   SSRSSTLTLPPGAPLTVSPDPSGTVLGGSIGPGGGLGVGMIGSGGVVQKVNASRAPSKHV  480

Query  1012  DEFELEIELESQRKRESGESRKMN  1035
             DEFELEIELESQRKRESGESRKMN
Sbjct  481   DEFELEIELESQRKRESGESRKMN  504


>emb|CED83978.1| hypothetical protein [Xanthophyllomyces dendrorhous]
Length=320

 Score =  614 bits (1584),  Expect = 0.0, Method: Compositional matrix adjust.
 Identities = 304/309 (98%), Positives = 304/309 (98%), Gaps = 0/309 (0%)

Query  1    MAPSLLFHSNIATYPSEPDSFLAVVRLPKSASLDSIVITPLGLRPFYYHQPDSNLDDGQT  60
            MAPSLLFHSNIATYPSEPDSFLAVVRLPKSASLDSIVITPLGLRPFYYHQPDSNLDDGQT
Sbjct  1    MAPSLLFHSNIATYPSEPDSFLAVVRLPKSASLDSIVITPLGLRPFYYHQPDSNLDDGQT  60

Query  61   PTRPAEIDLLFNARYPPSPLTQSATISTLPLIPSTKAPKGWKPNSLHKSTLSYNPFLRDS  120
            PTRPAEIDLLFNARYPPSPLTQSATISTLPLIPSTKAPKGWKPNSLHKSTLSYNPFLRDS
Sbjct  61   PTRPAEIDLLFNARYPPSPLTQSATISTLPLIPSTKAPKGWKPNSLHKSTLSYNPFLRDS  120

Query  121  DGAPPGIDSTSPLAIQQFEIGMPLDVTTTLVVLRCPFSIITLSLYGYPSTSLSAVADREE  180
            DGAPPGIDSTSPLAIQQFEIGMPLDVTTTLVVLRCPFSIITLSLYGYPSTSLSAVADREE
Sbjct  121  DGAPPGIDSTSPLAIQQFEIGMPLDVTTTLVVLRCPFSIITLSLYGYPSTSLSAVADREE  180

Query  181  SSMAGWETYLYNLSAGSQSNSSNNSFENVLLSVISARPNHPDRNATKETEKCGQYEEDMA  240
            SSMAGWETYLYNLSAGSQSNSSNNSFENVLLSVISARPNHPDRNATKETEKCGQYEEDMA
Sbjct  181  SSMAGWETYLYNLSAGSQSNSSNNSFENVLLSVISARPNHPDRNATKETEKCGQYEEDMA  240

Query  241  VDGFVLKEIADHPLQERLNEILKSLTHLIESSEEASTLHNEDTTEHLIRDTSSQPSSSIS  300
            VDGFVLKEIADHPLQERLNEILKSLTHLIESSEEASTLHNEDTTEHLIRDTSSQPSSSIS
Sbjct  241  VDGFVLKEIADHPLQERLNEILKSLTHLIESSEEASTLHNEDTTEHLIRDTSSQPSSSIS  300

Query  301  PEVCIELIL  309
            PE    L L
Sbjct  301  PEPSSPLYL  309


>gb|KIR41283.1| hypothetical protein I313_02403 [Cryptococcus gattii Ram5]
 gb|KIY56253.1| hypothetical protein I307_04358 [Cryptococcus gattii 99/473]
Length=559

 Score = 44.3 bits (103),  Expect = 0.71, Method: Compositional matrix adjust.
 Identities = 55/211 (26%), Positives = 87/211 (41%), Gaps = 63/211 (30%)

Query  1    MAPSLLFHSNI-ATYPSEPDSFLAVVRLPKSASLDSIVITPLGLRPFYYHQPDSNLDDGQ  59
            M P LL+HS+  + YP+   S L V+R      + S+ ITP G+R            DG 
Sbjct  1    MIPQLLYHSSSPSMYPTNTCSSLLVLRFSGPVRVSSVRITPEGVR----------CPDGS  50

Query  60   TPTRPAEI--DLLFNARYPPSPLTQSATISTLPLIPSTKAPKGWKPNSLHKSTLSYNPFL  117
              T PA+    LLFN              S+ P+            N+L  + ++YN  L
Sbjct  51   GVTYPAKWTGQLLFNIS------------SSNPV------------NALVSTNITYNAAL  86

Query  118  RDSDGAPPGIDSTSPLAIQQFEIGMPLDVTTTLVVLRCPFSIITLSLYGYP-------ST  170
               D                + I MP   T+ +++LR P   +++S+YGY        +T
Sbjct  87   HPVD----------------YPIDMPRGTTSRMMMLRAPVEKLSISVYGYTDEPPGGDAT  130

Query  171  SLSAVADREESSMAGWE---TYLYNLSAGSQ  198
               AV D++ S+    E   ++L+  +  SQ
Sbjct  131  QKDAVDDQKPSNTTSEEEDSSWLWRWAGDSQ  161


>gb|KIR26362.1| hypothetical protein I309_04696 [Cryptococcus gattii LA55]
 gb|KIR89975.1| hypothetical protein I304_06227 [Cryptococcus gattii CBS 10090]
 gb|KIR98702.1| hypothetical protein L804_04285 [Cryptococcus gattii 2001/935-1]
Length=559

 Score = 44.3 bits (103),  Expect = 0.72, Method: Compositional matrix adjust.
 Identities = 55/211 (26%), Positives = 87/211 (41%), Gaps = 63/211 (30%)

Query  1    MAPSLLFHSNI-ATYPSEPDSFLAVVRLPKSASLDSIVITPLGLRPFYYHQPDSNLDDGQ  59
            M P LL+HS+  + YP+   S L V+R      + S+ ITP G+R            DG 
Sbjct  1    MIPQLLYHSSSPSMYPTNTCSSLLVLRFSGPVRVSSVRITPEGVR----------CPDGS  50

Query  60   TPTRPAEI--DLLFNARYPPSPLTQSATISTLPLIPSTKAPKGWKPNSLHKSTLSYNPFL  117
              T PA+    LLFN              S+ P+            N+L  + ++YN  L
Sbjct  51   GVTYPAKWTGQLLFNIS------------SSNPV------------NALVSTNITYNAAL  86

Query  118  RDSDGAPPGIDSTSPLAIQQFEIGMPLDVTTTLVVLRCPFSIITLSLYGYP-------ST  170
               D                + I MP   T+ +++LR P   +++S+YGY        +T
Sbjct  87   HPVD----------------YPIDMPRGTTSRMMMLRAPVEKLSISVYGYTDEPPGGDAT  130

Query  171  SLSAVADREESSMAGWE---TYLYNLSAGSQ  198
               AV D++ S+    E   ++L+  +  SQ
Sbjct  131  QKDAVDDQKPSNTTSEEEDSSWLWRWAGDSQ  161


>gb|KIR69972.1| hypothetical protein I310_06292 [Cryptococcus gattii CA1014]
 gb|KNX50040.1| hypothetical protein CNBG_9253 [Cryptococcus gattii R265]
Length=559

 Score = 44.3 bits (103),  Expect = 0.75, Method: Compositional matrix adjust.
 Identities = 55/211 (26%), Positives = 87/211 (41%), Gaps = 63/211 (30%)

Query  1    MAPSLLFHSNI-ATYPSEPDSFLAVVRLPKSASLDSIVITPLGLRPFYYHQPDSNLDDGQ  59
            M P LL+HS+  + YP+   S L V+R      + S+ ITP G+R            DG 
Sbjct  1    MIPQLLYHSSSPSMYPTNTCSSLLVLRFSGPVRVSSVRITPEGVR----------CPDGS  50

Query  60   TPTRPAEI--DLLFNARYPPSPLTQSATISTLPLIPSTKAPKGWKPNSLHKSTLSYNPFL  117
              T PA+    LLFN              S+ P+            N+L  + ++YN  L
Sbjct  51   GVTYPAKWTGQLLFNIS------------SSNPV------------NALVSTNITYNAAL  86

Query  118  RDSDGAPPGIDSTSPLAIQQFEIGMPLDVTTTLVVLRCPFSIITLSLYGYP-------ST  170
               D                + I MP   T+ +++LR P   +++S+YGY        +T
Sbjct  87   HPVD----------------YPIDMPRGTTSRMMMLRAPVEKLSISVYGYTDEPPGGDAT  130

Query  171  SLSAVADREESSMAGWE---TYLYNLSAGSQ  198
               AV D++ S+    E   ++L+  +  SQ
Sbjct  131  QKDAVDDQKPSNTTSEEEDSSWLWRWAGDSQ  161


>gb|KIR34330.1| hypothetical protein I352_03574 [Cryptococcus gattii MMRL2647]
Length=559

 Score = 44.3 bits (103),  Expect = 0.76, Method: Compositional matrix adjust.
 Identities = 55/211 (26%), Positives = 87/211 (41%), Gaps = 63/211 (30%)

Query  1    MAPSLLFHSNI-ATYPSEPDSFLAVVRLPKSASLDSIVITPLGLRPFYYHQPDSNLDDGQ  59
            M P LL+HS+  + YP+   S L V+R      + S+ ITP G+R            DG 
Sbjct  1    MIPQLLYHSSSPSMYPTNTCSSLLVLRFSGPVRVSSVRITPEGVR----------CPDGS  50

Query  60   TPTRPAEI--DLLFNARYPPSPLTQSATISTLPLIPSTKAPKGWKPNSLHKSTLSYNPFL  117
              T PA+    LLFN              S+ P+            N+L  + ++YN  L
Sbjct  51   GVTYPAKWTGQLLFNIS------------SSNPV------------NALVSTNITYNAAL  86

Query  118  RDSDGAPPGIDSTSPLAIQQFEIGMPLDVTTTLVVLRCPFSIITLSLYGYP-------ST  170
               D                + I MP   T+ +++LR P   +++S+YGY        +T
Sbjct  87   HPVD----------------YPIDMPRGTTSRMMMLRAPVEKLSISVYGYTDEPPGGDAT  130

Query  171  SLSAVADREESSMAGWE---TYLYNLSAGSQ  198
               AV D++ S+    E   ++L+  +  SQ
Sbjct  131  QKDAVDDQKPSNTTSEEEDSSWLWRWAGDSQ  161


Lambda      K        H        a         alpha
   0.316    0.131    0.377    0.792     4.96 

Gapped
Lambda      K        H        a         alpha    sigma
   0.267   0.0410    0.140     1.90     42.6     43.6 

Effective search space used: 12717186230000


  Database: nr
    Posted date:  Sep 23, 2015 12:05 AM
  Number of letters in database: 26,053,659,533
  Number of sequences in database:  71,551,133


Matrix: BLOSUM62
Gap Penalties: Existence: 11, Extension: 1
Neighboring words threshold: 11
Window for multiple hits: 40
```
